# Electron Beam Therapy for Perforating Granuloma Annulare: A Case Report

**DOI:** 10.1159/cde/abvag010

**Published:** 2026-09-30

**Authors:** Shuo Li, Jinli Liu, Jinke Ma, Jun Tang

**Affiliations:** a Division of Life Sciences and Medicine, The Dermatology Department of The First Affiliated Hospital of USTC, University of Science and Technology of China, Hefei, PR China; b Department of Radiation Oncology, The 901st Hospital of the Joint Logistics Support Force of the Chinese People’s Liberation Army, Hefei, PR China

**Keywords:** Perforating granuloma annulare, Electron beam therapy, Dermatological radiotherapy, Case report

## Abstract

**Introduction:**

Perforating granuloma annulare (PGA) is a rare benign inflammatory dermatosis, accounting for merely 5% of all granuloma annulare (GA) cases. Standardized treatment regimens for refractory PGA are still unavailable, and there are sparse published reports evaluating electron beam therapy (EBT) for this specific skin disease.

**Case Presentation:**

We describe a 66-year-old Chinese woman with a 5-year history of refractory PGA affecting the right forearm, who showed no response to multiple conventional therapies. The patient initially received an EBT-based multimodal regimen, and subsequent EBT monotherapy was administered for residual lesions. After treatment, remarkable clinical improvement with near-complete resolution of PGA lesions was obtained. No disease recurrence or radiation-related adverse events were detected over the 10-month follow-up.

**Conclusion:**

Low-dose superficial EBT may be a promising, well-tolerated individualized therapeutic alternative for refractory PGA. Our findings provide supplementary real-world clinical evidence for research on this rare inflammatory dermatosis. Further clinical investigations are needed to validate the long-term efficacy of EBT and establish unified treatment protocols for PGA.

## Plain Language Summary

Perforating granuloma annulare (PGA) is an exceptionally rare, benign skin disorder that is often challenging to manage, particularly in cases where standard treatments have failed. We present the case of a 66-year-old woman with a 5-year history of treatment-resistant PGA affecting the right forearm, who experienced no benefit from multiple prior conventional therapies. The patient was treated with electron beam therapy (EBT), a form of targeted radiation. EBT was first administered as part of a multimodal treatment regimen, followed by EBT monotherapy for residual lesions. Following treatment, the patient’s lesions showed marked improvement with near-complete resolution. No disease recurrence or serious radiation-related adverse events were observed over the 10-month follow-up period. This case indicates that low-dose superficial EBT might be a safe, effective and individualized treatment option for patients with refractory PGA. Further prospective studies are needed to validate these findings and establish evidence-based care guidelines for this rare, clinically challenging skin disorder.

## Introduction

Perforating granuloma annulare (PGA), a rare subtype accounting for 5% of granuloma annulare (GA), is an etiologically unclear noninfectious granulomatous dermatosis [1]. Clinically, it presents as skin-colored or erythematous papules, with central crusts, scales, or umbilication. Although PGA is self-limiting, no specific therapy exists, and intervention is required for prolonged courses or recurrence to control symptoms and improve cosmesis [1, 2]. Electron beam therapy (EBT), a form of radiotherapy that employs high-energy electron beams generated by a linear accelerator, delivers precise superficial tissue irradiation while sparing deeper structures, making it particularly suitable for skin diseases [3]. EBT has demonstrated efficacy in managing conditions such as mycosis fungoides and keloids, with evidence supporting its role in reducing keloid recurrence rates, and it also exhibits favorable efficacy and a favorable safety profile in the management of a variety of benign cutaneous disorders [4–6]. However, few published studies have investigated the therapeutic efficacy of EBT in PGA. This report presents a case of PGA predominantly involving the right forearm, which achieved a favorable response following EBT.

## Case Presentation

A 66-year-old Chinese female presented with a 5-year progressive skin lesion on her right forearm, accompanied by pruritus, as well as vesicles and exudate at the lesion margins. Physical examination identified a well-demarcated erythematous plaque on the extensor surface of the right forearm, measuring approximately 5 × 10 cm. This plaque was marginally elevated, associated with multiple umbilicated papules, and exhibited keratinous material in the center and crusting on the surface; several smaller satellite lesions were scattered around this primary lesion (Fig. 1a). To confirm the diagnosis, skin biopsy specimens were collected from the peripheral margins of two well-demarcated plaques within the right forearm lesion. Histopathological analysis revealed inflammatory cell infiltration and collagen degeneration, with inflammatory cells and histiocytes forming a typical palisading granuloma surrounding the degenerated collagen (Fig. 1b, c). In addition, partial transepidermal elimination of degenerated collagen could be observed microscopically, with fragmented collagen penetrating and extruding through the epidermis (Fig. 1d, e). Supplementary Masson’s trichrome staining further validated this pathological feature (Fig. 1f). Combined assessment of all above histopathological findings and clinical cutaneous manifestations supported the diagnosis of PGA.

**Fig. 1.**
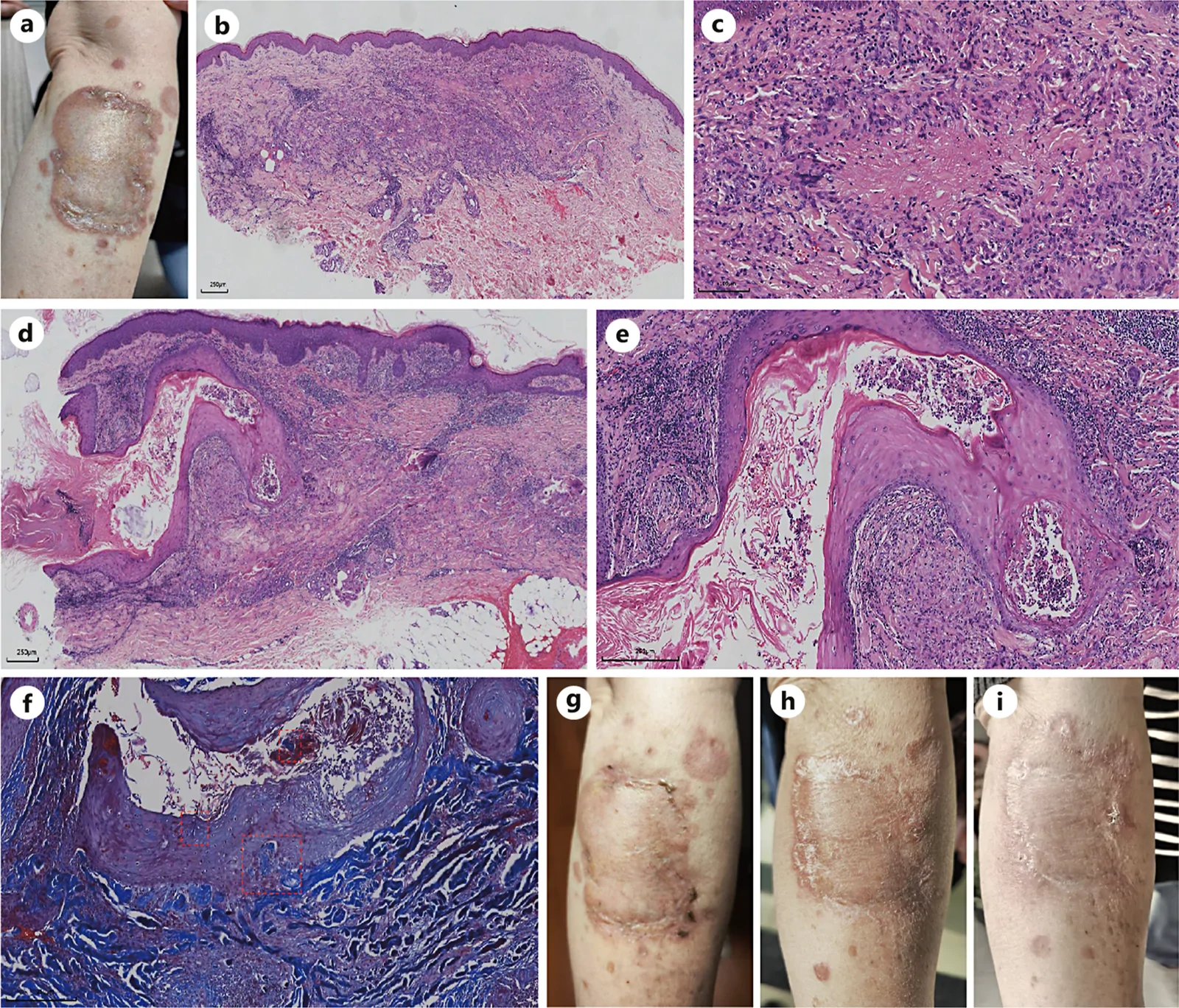
**a** Clinical manifestations of the patient’s skin lesions before treatment: 5 × 10 cm erythematous plaque with umbilicated papules, crusting and exudate. **b** Palisading granuloma: central collagen degeneration surrounded by palisaded histiocytes and inflammatory cells (×40). **c** Palisading granuloma: central collagen degeneration surrounded by palisaded histiocytes and inflammatory cells (×200). **d** Percutaneous phenomenon: dermal collagen degeneration with surrounding histiocytic and lymphocytic infiltration, and transepidermal elimination of degenerated collagen fibers (×40). **e** Percutaneous phenomenon: dermal collagen degeneration with surrounding histiocytic and lymphocytic infiltration, and transepidermal elimination of degenerated collagen fibers (×200). **f** Supplementary Masson’s trichrome staining (×200): Collagen components are stained blue. Degenerated collagen fibers penetrating the invaginated epidermis are marked within the red dashed box. **g** Clinical manifestations at 3 months after EBT combined therapy: significant reduction of lesion area, mild residual erythema, no exudate. **h** Follow-up at 7 months after EBT combined therapy: no recurrence, with mild residual erythema and local hypertrophy. **i** 3 months after EBT monotherapy: further reduction of residual lesion area, complete resolution of elevated margins/papules, erythema faded to near-normal skin tone, no pruritus or exudate.

A multimodal therapeutic regimen was initiated upfront, consisting of single-field local EBT (6 MeV electrons, 6 Gy per fraction, alternate days for 3 initial fractions, target depth 0.5 cm), a single intralesional injection of betamethasone (7 mg), oral tofacitinib (5 mg twice daily for 1 month), and topical corticosteroid ointment. Clinical response was assessed per standard dermatological metrics, including erythema and hypertrophy grading (0–4 scale), pruritus Visual Analog Scale (VAS; 0–10 scale), and exudate evaluation. At the 3-month follow-up (Fig. 1g), the erythema/hypertrophy grade decreased from 3 to 1, the pruritus VAS score declined from 7 to 2, and exudate resolved completely, with marked improvement in cutaneous lesions relative to baseline. At the 7-month follow-up, no lesion recurrence was noted, with only mild residual erythema and focal hypertrophy (erythema/hypertrophy grade 1 each) at the original lesional site (Fig. 1h); pruritus and exudate were absent. For these residual cutaneous changes, single-field local EBT monotherapy (same dosage and target depth as the initial regimen) was administered, alternating days for 2 fractions. At the 3-month follow-up after this second EBT course (10-month post-initial management; Fig. 1i), prominent clinical improvement was observed: erythema/hypertrophy grade resolved to 0 (with complete disappearance of marginal elevation and umbilicated papules), pruritus VAS score remained 0, and lesional erythema faded to near-normal skin tone. No recurrence or radiation-associated adverse events were documented in the EBT-irradiated field throughout the entire follow-up period.

## Discussion

In this case, EBT-based multimodal therapy achieved rapid and significant improvement in all objective and subjective clinical parameters of refractory PGA lesions in the early treatment phase, and EBT monotherapy for residual lesions also achieved notable clinical improvement. EBT was selected for this refractory case primarily due to its unique physical and clinical properties that align with PGA's pathophysiological features: its 6 MeV superficial irradiation with a 0.5 cm target depth delivers precise energy to the superficial dermis – where PGA's granulomatous inflammation and collagen degeneration occur – while sparing deeper subcutaneous tissue and vital organs; single-field EBT exerts localized action only on the affected lesion, avoiding systemic side effects associated with oral immunosuppressants or JAK inhibitors [3, 7]. The favorable clinical response is plausibly mediated by EBT's targeted suppression of local granulomatous inflammation and modulation of abnormal collagen turnover: ionizing radiation inhibits the proliferation and cytokine secretion of dermal lymphocytes and histiocytes, attenuating palisading granuloma formation; it also modulates collagen metabolism by inhibiting abnormal synthesis and promoting clearance of degenerated collagen, consistent with the resolution of transepidermal elimination observed in this patient. Notably, the low-dose superficial EBT administered (30 Gy total cumulative dose) differs substantially from high-dose radiotherapy for malignant diseases and has a well-documented favorable safety profile in dermatology – no acute or chronic radiation-related adverse events were observed in this case, consistent with its established use in other benign cutaneous disorders [4–7]. For this patient with refractory PGA lacking standardized treatment options, the clinical benefits of symptom relief and cosmetic improvement likely outweigh the minor potential risks of EBT exposure. These observational results imply that EBT could serve as a novel individualized treatment candidate for GA, especially its rare perforating subtype. However, evidence for the efficacy of EBT in PGA is currently limited to this single case report, with no dedicated clinical trials to date. Further prospective case series and small-scale clinical trials are therefore warranted to validate the clinical utility of low-dose EBT for refractory PGA and to establish evidence-based treatment protocols via comparisons with existing empirical therapeutic modalities using standardized clinical efficacy endpoints. Additionally, basic research into the molecular mechanism of EBT in modulating granulomatous inflammation and collagen metabolism in PGA is needed to strengthen the scientific foundation for this intervention. The CARE checklist supporting this case report has been provided as online supplementary material (for all online suppl. material, see https://doi.org/10.1159/cde/abvag010).

## Conclusions

This case report describes a clinical application of EBT for refractory PGA, supplementing limited real-world treatment data for this rare cutaneous disorder. In this patient with treatment-refractory PGA, low-dose superficial EBT delivered durable clinical benefit, whether used as part of a multimodal induction regimen or as monotherapy for residual disease. This case offers preliminary proof-of-concept evidence indicating that low-dose superficial EBT may be a viable, well-tolerated and individualized treatment alternative for refractory PGA patients unresponsive to conventional therapies, providing one optional intervention for this therapeutically challenging dermatosis.

## Statement of Ethics

Formal ethical approval was not required for this retrospective case report by the Medical Research Ethics Committee of the First Affiliated Hospital of University of Science and Technology of China in accordance with local institutional guidelines, as it solely utilized existing clinical data and imposed no additional patient risks. This study complied with our hospital’s ethical standards. Written informed consent was signed by the patient, who was fully informed and agreed to publish all relevant clinical records, skin images and pathological data in *Case Reports in Dermatology*. The signed consent original is archived in our department, with a scanned copy provided only for editorial audit. All personal identifiers are erased, and identifying features on clinical photos are masked. The patient was notified of open-access publication and may withdraw consent before online release.

## Conflict of Interest Statement

The authors have no conflicts of interest to declare.

## Funding Sources

This work was supported by the National Natural Science Foundation of China (Grant No. 82273516). The funding was utilized to pay for manuscript publication fees and partial examination and treatment costs of the study patients. The funder was not involved in study design, data acquisition and analysis, manuscript drafting, or the decision to publish.

## Author Contributions

Shuo Li: data collection, clinical follow-up, and manuscript drafting; Jinli Liu: clinical assessment, histopathological analysis, and manuscript revision; Jinke Ma: radiation therapy regimen design, treatment guidance, and manuscript review; Jun Tang: conceptualization, study supervision, final manuscript revision, and approval of the published version.

## Data Availability

The data that support the findings of this study are not publicly available due to patient privacy but are available from the corresponding author upon reasonable request.
